# A promoter SNP rs4073T>A in the common allele of the interleukin 8 gene is associated with the development of idiopathic pulmonary fibrosis via the IL-8 protein enhancing mode

**DOI:** 10.1186/1465-9921-12-73

**Published:** 2011-06-08

**Authors:** Mi-Hyun Ahn, Byung-Lae Park, Shin-Hwa Lee, Sung-Woo Park, Jong-Sook Park, Do-Jin Kim, An-Soo Jang, Jai-Soung Park, Hwa-Kyun Shin, Soo-Taek Uh, Yang-Ki Kim, Young Whan Kim, Sung Koo Han, Ki-Suck Jung, Kye Young Lee, Sung Hwan Jeong, Jeong Woong Park, Byoung Whui Choi, In Won Park, Man Pyo Chung, Hyoung Doo Shin, Jin Woo Song, Dong Soon Kim, Choon-Sik Park, Young-Soo Shim

**Affiliations:** 1 Div. of Allergy and Respiratory Medicine, Dept. of Internal Medicine, Soonchunhyang Univ. Bucheon Hospital, 1174, Jung-dong, Wonmi-gu, Bucheon, 420-020, Korea; 2 Dept. of Genetic Epidemiology, SNP-Genetics Inc., B-1407, WooLim Lion's Valley, 371-28 Gasan-Dong, Geumcheon-Ku, Seoul, 153-803, Korea; 3 Div. of Radiology, Soonchunhyang Univ. Bucheon Hosp., 1174, Jung-Dong, Wonmi-Gu, Bucheon, Gyeonggi-Do, 420-020, Korea; 4 Div. of Thoracic and Cardiovascular Surgery, Soonchunhyang Univ. Bucheon Hosp., 1174, Jung-Dong, Wonmi-Gu, Bucheon, Gyeonggi-Do, 420-020, Korea; 5 Div. of Allergy and Respiratory Medicine, Soonchunhyang Univ. Seoul Hosp., 657-58, Hannam-dong, Yongsan-gu, Seoul, 140-743, Korea; 6 Dept. of Internal Medicine, Seoul National Univ. Hosp., 28 Yongon-dong, Seoul, Korea; 7 Dept. of Respiratory and Critical Care Medicine, Hallym Univ., Korea; 8 Dept. of Internal Medicine, College of Medicine, Dankook Univ., Cheonan, Korea; 9 Div. of Pulmonary Medicine, Dept. of Internal Medicine, Gachon Medical School Gil Medical Center, Korea; 10 Department of Internal Medicine, Chung Ang University College of Medicine, Seoul, Korea; 11 Div. of Pulmonary and Critical Care Medicine, Samsung Medical Center, Sungkyunkwan Univ. School of Medicine, Seoul, Korea; 12 Dept. of Life Science, Sogang Univ., Sinsu-dong, Mapo-gu, Seoul, 121-742, Korea; 13 Div. of Pulmonary and Critical Care Medicine, Asan Medical Center, Univ. of Ulsan, Asanbyungwon-gil, Songpa-gu, Seoul, 138-736, Korea; 14 Dept. of Medicine, Armed Force Capital Hospital, Bundang-gu, Seongnam-si, Kyonggi-do, Korea

## Abstract

**Background:**

Interleukin-8 (IL-8) is a potent chemo-attractant cytokine responsible for neutrophil infiltration in lungs with idiopathic pulmonary fibrosis (IPF). The IL-8 protein and mRNA expression are increased in the lung with IPF. We evaluated the effect of single nucleotide polymorphisms (SNPs) of the IL-8 gene on the risk of IPF.

**Methods:**

One promoter (rs4073T>A) and two intronic SNPs (rs2227307T>G and rs2227306C>T) of the IL-8 genes were genotyped in 237 subjects with IPF and 456 normal controls. Logistic regression analysis was applied to evaluate the association of these SNPs with IPF. IL-8 in BAL fluids was measured using a quantitative sandwich enzyme immunoassay, and promoter activity was assessed using the luciferase reporter assay.

**Results:**

The minor allele frequencies of rs4073T>A and rs2227307T>G were significantly lower in the 162 subjects with surgical biopsy-proven IPF and 75 subjects with clinical IPF compared with normal controls in the recessive model (OR = 0.46 and 0.48, *p *= 0.006 and 0.007, respectively). The IL-8 protein concentration in BAL fluids significantly increased in 24 subjects with IPF compared with 14 controls (*p *= 0.009). Nine IPF subjects homozygous for the rs4073 T>A common allele exhibited higher levels of the IL-8 protein compared with six subjects homozygous for the minor allele (*p *= 0.024). The luciferase activity of the rs4073T>A common allele was significantly higher than that of the rs4073T>A minor allele (*p *= 0.002).

**Conclusion:**

The common allele of a promoter SNP, rs4073T>A, may increase susceptibility to the development of IPF via up-regulation of IL-8.

## Introduction

Idiopathic pulmonary fibrosis (IPF) is a devastating disease of the idiopathic interstitial pneumonia family. It predominantly affects the lung parenchyma and is characterized by progressive dyspnea and worsening lung function [1]. Although the pathogenesis of IPF is largely unknown, a current hypothesis suggests aberrant wound healing of ongoing alveolar epithelial injury and repair associated with the formation of patchy fibroblast-myofibroblast foci, which evolve to fibrosis [2,3]. The processes of inflammation and fibrosis likely involve an interaction between environmental triggers and genetic background [2]. Supporting evidence for the genetic background for pulmonary fibrosis is the familial occurrence, as seen in familial IPF [4]. However, the nature of the genetic basis for sporadic IPF has not been evaluated due to low disease incidence. Recent reports suggest that genetic polymorphisms of putative candidate genes contribute to the development of lung fibrosis [5-7].

Characteristic of IPF is neutrophilia of the bronchoalveolar lavage fluid. The recruitment and activation of neutrophils plays a fundamental role in the development of lung injury, which precedes aberrant wound repair in the pathogenesis of IPF[3]. Interleukin-8 (IL-8) acts as a potent chemoattractant for neutrophils [8]. The IL-8 protein and mRNA expression are increased in the BAL fluid and the alveolar macrophages of patients with IPF [9]. An animal study also confirmed the role of IL-8 in pulmonary fibrosis by demonstrating that bleomycin-induced lung fibrosis is attenuated by the neutralization of IL-8[10]. In addition to promoting inflammation, IL-8 has angiogenic activity[11,12]. Thus, genetic alterations of IL-8 may be related to the development of IPF.

In humans, the gene encoding IL-8 is located on chromosome 4q12-q21 and consists of four exons and three introns [13]. Polymorphisms of IL-8 are associated increased risk of developing various cancers [14]. SNPs within IL8 have been reported as candidates for cystic fibrosis lung disease, a neutrophil-dominant inflammatory lung disease like IPF [15]. Although a previous study reported no association between IPF risk and these SNPs [16], the study had a small sample size of 71 patients with IPF including 31 surgical biopsy-proven cases. Thus, a study with a relatively large sample size was needed to examine the genetic effect of polymorphisms of the IL-8 gene on the risk of IPF. We genotyped and compared the frequencies of three SNPs of the IL-8 genes in 237 subjects with IPF and 456 normal controls and evaluated their association with the development of IPF, as well as performed functional validation.

## Methods

### Study subjects

Subjects with IPF were recruited from the Korean Cohort of Interstitial Lung Disease. The study population comprised 237 patients with IPF recruited from January 1984 to November 2004 from eight university hospitals. Normal (control) subjects (n = 456) were the spouses of the patients or volunteers from the general population. Control subjects were at least 50 years old, had no respiratory symptoms, exhibited normal FVC and FEV1 (>75% of the predicted value), and normal findings on a simple chest posterior-anterior view x-ray. The diagnosis of IPF was based on an international consensus statement by ATS/ERS with compatible findings via surgical lung biopsy (*n *= 162) or using radio-clinical criteria (*n *= 75), i.e., the presence of clinical, functional, and high-resolution computed tomography patterns strongly consistent with IPF. None of the patients with IPF had any evidence of the underlying collagen vascular diseases clinically or by laboratory diagnosis. The institutional review board by Soonchunhyang University hospital for human studies approved the protocol, and informed written consent was obtained from all subjects.

### Genotyping with fluorescence polarization detection

To genotype polymorphic sites, primers and probes were designed for TaqMan^® ^17. Primer Express (Applied Biosystems, Foster, CA, USA) was used to design both the PCR primers and the MGB TaqMan probes. One allelic probe was labeled with the FAM dye and the other was labeled with fluorescent VIC dye. The PCRs were run on the TaqMan Universal Master mix without UNG (Applied Biosystems), with a PCR primer concentration of 900 nM and a TaqMan MGB-probe concentration of 200 nM. The reactions were carried out in a 384-well format in a total reaction volume of 50 ul using 20 ng of the genomic DNA. The plates then were placed in a thermal cycler (PE 9700, Applied Biosystems) and heated to 50°C for 2 min and 95°C for 10 min followed by 40 cycles of 95°C for 15 sec and 60°C for 1 min. The TaqMan assay plates were then transferred to a Prism 7900HT instrument (Applied Biosystems), which measured the fluorescence intensity in each well of the plate. The fluorescence data files from each plate were analyzed using automated software (SDS 2.1). Detailed information concerning the primers is presented in additional file 1, table S1.

### Bronchoalveolar lavage and enzyme immunoassay of IL-8

BAL had been performed in the most affected lobe by computed tomography in the 24 subjects without any immunosuppressive therapy and in the right middle lobe of 14 normal controls, as described previously[17]. The supernatant was separated from cell pellets by centrifugation at 500 × *g *for 5 minutes. IL-8 in BAL fluids was measured using a quantitative sandwich enzyme immunoassay kit (BD Pharmingen, San Diego, CA, USA). The lower limit of detection for IL-8 was 15.6 pg/mL. Values below this limit were assumed to be 0 pg/mL for the statistical analysis. The inter- and intra-assay coefficients of variance were below 10%. Protein concentration of BAL samples was measured for standardization using a micro BCA protein assay kit (Pierce, Rockford, IL, USA).

### Assessing promoter activity using the luciferase reporter assay

The promoter region of IL-8 was amplified using PCR. The genomic DNA fragment was isolated from B cell lines of the IPF subjects using a genomic DNA preparation kit (Gentra, Ipswich, MA, USA). The first PCR product was amplified using the following primers: forward; 5'-TGCCTTTGGAAGATTCTGCT-3', reverse; 5'-GCCAGCTTGGAAGTCATGTT-3'. The primary PCR reaction mixture was diluted and used as a template for a nested PCR reaction using the nested primers containing restriction enzyme sequences (forward; 5'-ACTGGTACC(KpnI)ACATTACTCAGAAA-3', reverse; 5'-CCTACGCGT(MluI)GTCTCTGAAAGTTTG-3') for construction of the IL8 reporter plasmid. The amplified fragment of the promoter region of the IL8 gene (-79 to -743 bp from the transcription start site) was cloned using the pGEM-T easy vector system (Promega Co. Madison, WI, USA), was ligated with pGL-3 basic Luc+ reporter vector (Promega). Cloned DNA sequences were determined by a DNA direct-sequencing service (Genotech, Daejeon, Korea). One day before transfection, 293 T cells were seeded at 5 × 10^5 ^cells per well (6-well plate) in 2 ml with 10% FBS. A 2-μg aliquot of the IL8-pGL3 basic constructor plasmid and 50 ng of PSV-galactosidase reporter vector (Promega, transfection parameter) were diluted in 250 μl OptMEM (GIBCO BRL, Burlington, MD, USA) without serum. The 4 μl of lipofectamine 2000 (recommended DNA ug: lipofectamine ul = 1:2, Invitrogen, Carlsbad, CA) was diluted in 250 ul OptMEM (GIBCO BRL) per well. The diluted DNA was combined with the diluted lipid (total volume 500 μl per well). Then, 500 μl of transfection complex was added, and the cells were incubated at 37°C with 5% CO2 in humidified air for 48 h. β-galactosidase activity was measured by ortho-nitrophenyl-D-galactopyranoside (ONPG) hydrolysis using β-Gal Assay kit (Promega). The cells were solubilized by scraping with 400 μl of cell lysis buffer of Luciferase Assay System kit (Promega). Luciferase activity was measured using the Luciferase Assay System and luminometer (VICTOR3, Perkinelmer, Waltham, MA, USA). And the relative luciferase activity was normalized to the protein concentration and β-galactosidase activity.

### Statistics

We applied widely used measures of linkage disequilibrium to all pairs of biallelic loci: Lewontin's D' (|D'|) [18] and *r^2^*. Haplotypes of each individual were inferred using the PHASE algorithm (ver. 2.0) developed by Stephens *et al*. [19]. The genotype and haplotype distributions were analyzed using logistic regression models with age (continuous value), gender (male = 0, female = 1), smoking status (non-smoker = 0, ex-smoker = 1, smoker = 2), atopy (absence = 0, presence = 1), and BMI as covariates. Cox models were used for calculating relative hazards and P-values controlling age, sex and smoking status[20]. Mantel-Haenszel chi-square (MHC) tests were used to test for trend in the categorical analysis. The data were managed and analyzed using SAS version 9.1 (SAS Inc., Cary, NC, USA). Statistical power of single associations was calculated with false-positive rate of 5% and four given MAFs and sample sizes and assuming a relative risk of 1.5, using PGA (Power for Genetic Association Analyses) software [21].

## Results

### Clinical profiles of study subjects

Clinical profiles of the study subjects are summarized in Table 1. In total, 237 subjects with IPF and 456 normal controls were recruited. Age and sex ratios of normal controls were similar to those of the subjects with IPF. The 162 subjects with biopsy-proven IPF and the 75 subjects with clinical IPF had similar age and sex ratios. The frequency of current smokers and ex-smokers were higher in the subjects with both biopsy-proven IPF and clinical IPF compared with that in normal controls. The patients with IPF had a significant reduction in FVC when compared with normal control subjects (*p *< 0.01). The subjects with biopsy-proven IPF and those with clinical IPF had the comparable impairment of FVC and DLCO.

**Table 1:** Clinical profiles of study subjects

Description	Normal controls	IPF	Clinical-IPF
N	456	162	75
Age, yr (range)	62 (50-87)	58 (41-83)	66 (47-83)
Sex (male/female)	278/178	112/50	51/24
Current Smoker (%)/Ex-smoker (%)	13.8/14.4	28.4/30.2	24.0/28.0
FVC % pred.	98.70 ± 16.73	72.56 ± 17.37	70.94 ± 17.28
DLCO % pred.	ND	66.60 ± 19.51	60.71 ± 22.05

IPF: Idiopathic pulmonary fibrosis

FVC: forced expiratory vital capacity

DLCO: Carbon Monoxide Diffusing Capacity

pred.: prediction

ND: not determined

### Association of SNPs within the *IL8 *gene with development of IPF

One promoter SNP (rs4073T>A) and two intronic SNPs (rs2227307T>G and rs2227306C>T) within the *IL8 *gene were genotyped in IPF patients and normal subjects (see Additional file 2, figure S1). Frequencies and heterozygosities of the SNPs are presented in additional file 3, table S2. Genotype distributions of the SNPs were in Hardy-Weinberg equilibrium (*p *< 0.05). The LDs were calculated, and haplotypes of IL8 polymorphisms were constructed (see Additional file 2, figure S1 B and C). Three major haplotypes with over 5% of MAF were detected. However, IL8-ht1 and IL8-ht2 were not analyzed due to their equivalency with IL8 rs4073 and L8 rs2227306, respectively. IL8 rs4073 and rs2227307 were significantly associated with a decreased risk of developing IPF and clinical IPF in the recessive model (OR = 0.46 and OR = 0.48, *p *= 0.006 and *p *= 0.007, respectively; Table 2). The minor allele frequencies of rs4073T>A and rs2227307T>G were significantly lower in the subjects with IPF compared with that in normal controls (33.7% vs. 36.8% and 33.6% and 36.7%, respectively). When the study subjects were stratified by gender, the association of the two SNPs was restricted to male gender (Table 3).

**Table 2:** The association of IL8 SNPs with the risk of idiopathic pulmonary fibrosis (IPF)

rs	s	Distribution		Codominant			Dominant			Recessive			MAF		Statistical power
		
		Case	NC	OR(95%CI)	P	Pcorr	OR(95%CI)	P	Pcorr	OR(95%CI)	P	Pcorr	Case	NC	
rs4073	T	87(41.63%)	191(42.16%)												
	AT	103(49.28%)	191(42.16%)	0.84(0.65-1.08)	0.17	0.22	1.00(0.71 -1.42)	0.99	1	**0.46(0.26-** **0.80)**	**0.006**	**0.008**	0.337	0.368	82.2%
	A	19(9.09%)	71(15.67%)												
rs2227307	T	94(42.34%)	191(42.35%)												
	GT	107(48.20%)	189(41.91%)	0.83(0.65-1.06)	0.14	0.19	0.97(0.69 -1.37)	0.87	1	**0.48(0.28-** **0.82)**	**0.007**	**0.009**	0.336	0.367	93.5%
	G	21(9.46%)	71(15.74%)												
rs2227306	C	103(48.13%)	216(47.58%)												
	CT	95(44.39%)	196(43.17%)	0.92(0.70-1.19)	0.51	0.67	0.97(0.69 -1.36)	0.84	1	0.70(0.37- 1.30)	0.26	0.34	0.297	0.308	79.9%
	T	16(7.48%)	42(9.25%)												
IL8_ht3	-/-	175(91.15%)	398(87.86%)												
	ht3/-	16(8.33%)	55(12.14%)	0.65(0.37-1.16)	0.15	0.19	0.61(0.34 -1.10)	0.10	0.13	.	.	.	0.047	0.061	35.6%
	ht3/ht3	1(0.52%)	0(0.00%)												

* Logistic models were used for calculating odd ration and p-values in recessive model controlling for age, sex, and smoking status as covariates.

NC: normal controls

MAF: Minor allele frequency

**Table 3:** The association of IL8 SNPs with the risk of idiopathic pulmonary fibrosis (IPF) by gender

**Male**				**Female**			
	
**MAF**		OR(95%CI)*	P*	**MAF**		OR(95%CI)*	P*
	
IPF and Clinical IPF (n = 152)	NC (n = 178)			IPF and Clinical IPF (n = 70)	NC (n = 276)		
	
0.340	0.394	**0.51 (0.26-0.97)**	**0.04**	0.331	0.351	0.53 (0.18-1.57)	0.25
0.336	0.394	**0.49 (0.26-0.93)**	**0.03**	0.336	0.350	0.57 (0.20-1.64)	0.30
0.288	0.326	0.83 (0.39-1.75)	0.62	0.316	0.297	0.64 (0.19-2.24)	0.49
0.064	0.071	.	.	0.008	0.054	.	.

* Logistic models were used for calculating odd ration and p-values in recessive model controlling for age, sex, and smoking status as covariates.

NC: normal controls

MAF: Minor allele frequency

### Association of rs4073T>A within the *IL-8 *gene with IL-8 protein levels in BAL fluids

**Figure 1:**
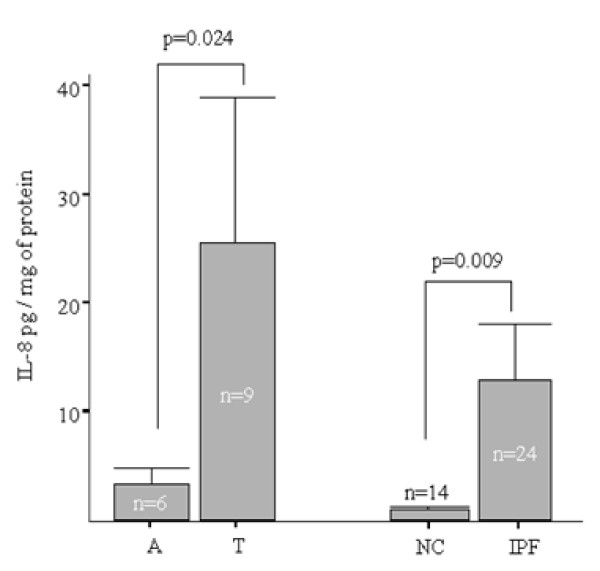
**Levels of IL-8 protein of in BAL fluid collected from normal controls and subjects with IPF**. NC: normal controls, IPF: Surgical IPF, A: IPF subjects having rs4073 *TT *alleles, T: IPF subjects having rs4073 *AA *alleles. Levels of IL-8 protein were normalized with BAL protein concentration.

The amount of IL-8 protein was measured in BAL fluids from 24 subjects with IPF and 14 NC. IL-8 concentrations were significantly increased in IPF patients compared with NC (9.24 ± 1.11 pg/mg of protein vs. 1.71 ± 0.27 pg/mg of protein, *p *= 0.009, Figure 1). A total of 15 subjects with IPF were genotyped, and the subjects with IPF exhibiting rs4073T>A, a common allele homozygote, had a higher level of IL-8 protein (27.01 ± 3.45 pg/mg of protein) than of minor allele homozygotes (2.35 ± 0.46 pg/mg of protein, *p *= 0.024). The IL-8 concentration in BAL fluids did not differ among individuals with the rs2227307T>G genotype (data, not shown).

### Comparison of promoter activity between TT and AA alleles of the IL8 promoter rs4073T>A

**Figure 2:**
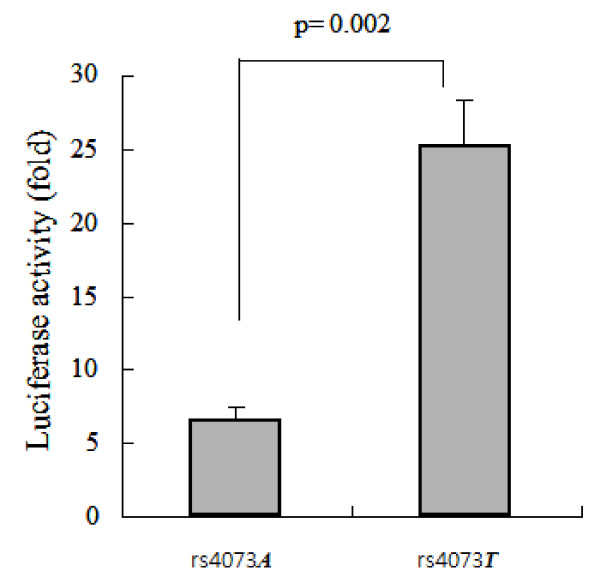
**Comparison of luciferase activity between rs40730*TT *and rs40703*AA *alleles**. The luciferase activity adjusted by pGL3 basic vector and the yield of DNA transfection adjusted using pSV-b-galactosidase (+) vector and ONPG activity.

Given that the rs4073*T>A *is located in the promoter region, we investigated the promoter activity of rs4073*T>A *using luciferase reporter assay. The luciferase activity was adjusted by pGL3 basic vector, and the yield of DNA transfection adjusted using pSV-b-galactosidase (+) vector and ONPG activity. The luciferase activity of the rs4073*T>A TT *allele was significantly higher than that of the rs4073*T>A AA *allele (25.2 ± 2.8 vs. 6.8 ± 0.7, *p *= 0.002, Figure 2).

## Discussion

Our logistic regression analysis of a case-control study determined that the *IL8 *rs4073T*>A *and rs2227307T>G SNPs from the promoter region are associated with development of IPF. The frequencies of the minor allele of the two SNPs were significantly decreased in IPF subjects compared with normal controls. These are the first data to indicate that the common alleles may increase susceptibility to development of IPF. Several reports have shown a relationship between IL8 gene polymorphisms and human lung diseases [22-26]. Two SNPs in the *IL8 *genes (rs4073 and rs2227307) were evaluated in patients with systemic sclerosis with (n = 78) or without fibrosing alveolitis (n = 50), those with cryptogenic fibrosing alveolitis (n = 71), and normal healthy subjects in the UK [16]. These study reported no association of the SNPs of *IL8 *with the risk of pulmonary fibrosis. The discrepancy between ours and the previously reported results may be due to the small study population in the previous study[16] or to ethnicity differences between study cohorts, as the minor allele frequency of rs4073T>A was 33.7% in our study subjects with IPF, whereas it was 56% in the UK study. Interestingly, the rs4073T>A polymorphism has recently been reported to be a risk factor of other lung diseases, including bronchial asthma [23] and bronchiolitis, caused by respiratory syncytial virus [22,24]. In addition, Hillian AD and coworkers reported an association of the rs 4073 T>A and cystic fibrosis when the analysis was restricted to male subjects. In the present study, the SNP was also significantly associated with IPF restricted to male gender[15]. This data suggest that the SNP may have a genetics effect on IL-8 gene expression in male gender, but not in female gender. We could not explain the restriction of the SNP to male gender. The location of IL-8 is in chromosome 4q13-q21, and the transcription factor supposed to bind to the SNP: eEF1A1 is in chromosome 6q14.1. Plasma IL-8 levels were reported to be similar in the subjects with male or female gender following sever trauma [15]. Further study on the association restricted to male would be performed.

We did not validate the association between the SNPs of the IL-8 gene in an independent replication population. We evaluate the effect of the SNP on IL-8 gene or protein expression instead. We measured IL-8 protein concentrations in the lung. IL-8 protein was increased in the BAL fluids of patients with IPF compared with normal controls. The IL-8 protein level in BAL fluid was significantly increased in the subjects with IPF having the common allele of rs4073T*>A *compared to those with the minor allele. This result indicates that the rs4073T*>A *allele within the promoter may result in increased IL-8 production when compared with the minor allele.

The promoter activity was examined using a luciferase reporter vector, and the promoter activity of the rs4073*T>A TT *allele was significantly stronger than that of the rs4073*T>A AA *allele. This is in accordance with a previous study, which reported that the rs4073*T>A TT *allele exhibited 2- to 5-fold stronger transcriptional activity than did the rs4073*T>A AA *counterpart [27]. Given that high IL-8 concentrations in BAL fluid were associated with the common allele of rs4073 T>A in the present study, our luciferase data confirm that the rs4073 *T *allele on the promoter may enhance the IL-8 transcription compared with the rs4073 A allele. Putative transcription factor binding sites in the promoter of the IL8 gene were searched using the TFSEARCH and TESS websites. The candidate binding protein for the transcription of IL8 at rs4073 was eEF1A1 (see Additional file 4, figure S2). The eEF1A family consists of two members, eEF1A1 and eEF1A2 [28]. Thus, eEF1A1 may regulate the activation and production of IL-8 as a transcription enhancer or inducer; this is a topic for future research.

In summary, we evaluated the genetic effect of IL-8 gene polymorphisms on the risk of IPF using a relatively large size population of subjects with IPF and normal controls. Logistic regression analysis demonstrated that the minor allele frequencies of rs4073T>A was significantly lower in the subjects with IPF compared with that in normal controls. The subjects with IPF homozygous for the rs4073*T>A *common allele exhibited significantly higher IL-8 protein concentrations in BAL fluids and enhanced luciferase activities compared with those homozygous for the rare allele. This study shows that the IL8 rs4073 T allele is significantly associated with an increased risk of IPF in the Korean population and this effect may result from the up-regulation of IL-8 protein synthesis in the lung. Our results may provide the clue of the genetic contribution to the pathogenesis of IPF.

## Abbreviations list

(IPF): Idiopathic pulmonary fibrosis; (IL-8): Interleukin-8; (ONPG): ortho-nitrophenyl-D-galactopyranoside; (|D'|): Lewontin's D'; (MHC): Mantel-Haenszel chi-square;

## Competing interests

The authors declare that they have no competing interests.

## Authors' contributions

MHA performed all experimental steps; BLP, SHL, and HDS analyzed statistics and wrote the manuscript; SWP, JSP, DJK and ASJ provided experimental assistance; JSP, HKS, SU, YK, YWK, SKH, KSJ, KYL, SHJ, JWP, BWC, IWP, MPC, JWS, DSK and YSS supervised this project; CSP conceptualized of the study and wrote the first draft of the manuscript. All authors read and approved the final manuscript.

The authors thank the editors from textcheck.com, both native speakers of English, for their proofreading for grammar and typographic errors. For a certificate, see http://www.textcheck.com/certificate/ox3Vhg.

## Supplementary Material

Additional file 1 **The fluorescence labeled allelic probe for amplification of IL8, IL8RA and IL8RB genes**. The data provided represent the probe for amplification of IL8, IL8RA and IL8RB genes. Click here for file

Additional file 2 **SNPs on the map of the IL8 gene, linkage disequilibrium, and haplotypes of IL8 genes**. The figure provided represent the map of the IL8 gene, linkage disequilibrium, and haplotypes of IL8 genes. Click here for file

Additional file 3 **The Minor allele frequency (MAF), Heterozygosity, Hardy-Weinberg equilibrium (HWE) of IL8 gene polymorphisms**. The data provided represent the MAF, HWE of IL8 gene polymorphisms. Click here for file

Additional file 4 **The candidate binding protein for the transcription of IL8 at rs4073**. The figure provided represent the putative transcription factor binding sites in the promoter of the IL8 gene. Click here for file
