# Supplementary material for: A promoter SNP rs4073T>A in the common allele of the interleukin 8 gene is associated with the development of idiopathic pulmonary fibrosis via the IL-8 protein enhancing mode
Source: Respir Res. 2011 Jun 8;12(1):73. doi: 10.1186/1465-9921-12-73 (PMC3141418; doi:10.1186/1465-9921-12-73)
Supplement: Additional file 1 — The fluorescence labeled allelic probe for amplification of IL8, IL8RA and IL8RB genes. The data provided represent the probe for amplification of IL8, IL8RA and IL8RB genes. [file 1465-9921-12-73-S1.DOC]

Supplementary table 1. The fluorescence labeled allelic probe for amplification of IL8, IL8RA and IL8RB genes

| Gene | SNP |  | Sequence (5' -> 3') |
| --- | --- | --- | --- |
|  |  |  |  |
| *IL8* | rs4073T>A | Forward Primer | TCTGTCACATGGTACTATGATAAAGTTATCTAGAAAT |
|  |  | Reverse Primer | ACGGAGTATGACGAAAGTTTTCTTTGA |
|  |  | VIC | TTGGTGAATTATCAA**A**TGTAT |
|  |  | FAM | TTGGTGAATTATCAA**T**TGTAT |
|  |  |  |  |
|  | rs2227307T>G | Forward Primer | CAGAATATAATCTTAGCAGTCAATT |
|  |  | Reverse Primer | TGAGGCTTGTCAATGAAAT |
|  |  | extension | TGATCAATATAGATATTCTGCTTTTATAATTTATACCA |
|  |  |  |  |
|  | rs2227306C>T | Forward Primer | TTGACCAGATAAAAATACCATGA |
|  |  | Reverse Primer | CCTAGCCCTTGACCTCAGT |
|  |  | extension | ATGATAAAACAGTCATAACTGACAACATTGAAC |
