# Supplementary material for: A promoter SNP rs4073T>A in the common allele of the interleukin 8 gene is associated with the development of idiopathic pulmonary fibrosis via the IL-8 protein enhancing mode
Source: Respir Res. 2011 Jun 8;12(1):73. doi: 10.1186/1465-9921-12-73 (PMC3141418; doi:10.1186/1465-9921-12-73)
Supplement: Additional file 2 — SNPs on the map of the IL8 gene, linkage disequilibrium, and haplotypes of IL8 genes. The figure provided represent the map of the IL8 gene, linkage disequilibrium, and haplotypes of IL8 genes. [file 1465-9921-12-73-S2.DOC]

**Supplementary figure 1.** SNPs on the map of the IL8 gene, linkage disequilibrium, and haplotypes of IL8 genes. **(A)** The human IL8 gene is located at 4q12–21. It consists of four exons, and the SNPs of the IL8 gene are located in the promoter and intron region. Bar = 500-base pair. (B) Haplotypes of IL8 gene. (C) LDs among IL8 polymorphisms.
