# Supplementary material for: A promoter SNP rs4073T>A in the common allele of the interleukin 8 gene is associated with the development of idiopathic pulmonary fibrosis via the IL-8 protein enhancing mode
Source: Respir Res. 2011 Jun 8;12(1):73. doi: 10.1186/1465-9921-12-73 (PMC3141418; doi:10.1186/1465-9921-12-73)
Supplement: Additional file 3 — The Minor allele frequency (MAF), Heterozygosity, Hardy-Weinberg equilibrium (HWE) of IL8 gene polymorphisms. The data provided represent the MAF, HWE of IL8 gene polymorphisms. [file 1465-9921-12-73-S3.DOC]

Supplementary table 2. The Minor allele frequency (MAF), Heterozygosity, Hardy-Weinberg equilibrium (HWE) of IL8 gene polymorphisms

| Gene | Loci | Genotype | | |  | Frequency | Heterozygosity | HWE |
| --- | --- | --- | --- | --- | --- | --- | --- | --- |
| IL8 | rs4073 | T | AT | A | N | 0.358 | 0.460 | 0.384 |
|  |  | 278 | 294 | 90 | 662 |  |  |  |
|  | rs2227307 | T | GT | G | N | 0.357 | 0.459 | 0.281 |
|  |  | 285 | 296 | 92 | 673 |  |  |  |
|  | rs2227306 | C | CT | T | N | 0.305 | 0.424 | 0.466 |
|  |  | 319 | 291 | 58 | 668 |  |  |  |
|  |  |  |  |  |  |  |  |  |
