# Supplementary material for: A promoter SNP rs4073T>A in the common allele of the interleukin 8 gene is associated with the development of idiopathic pulmonary fibrosis via the IL-8 protein enhancing mode
Source: Respir Res. 2011 Jun 8;12(1):73. doi: 10.1186/1465-9921-12-73 (PMC3141418; doi:10.1186/1465-9921-12-73)
Supplement: Additional file 4 — The candidate binding protein for the transcription of IL8 at rs4073. The figure provided represent the putative transcription factor binding sites in the promoter of the IL8 gene. [file 1465-9921-12-73-S4.DOC]

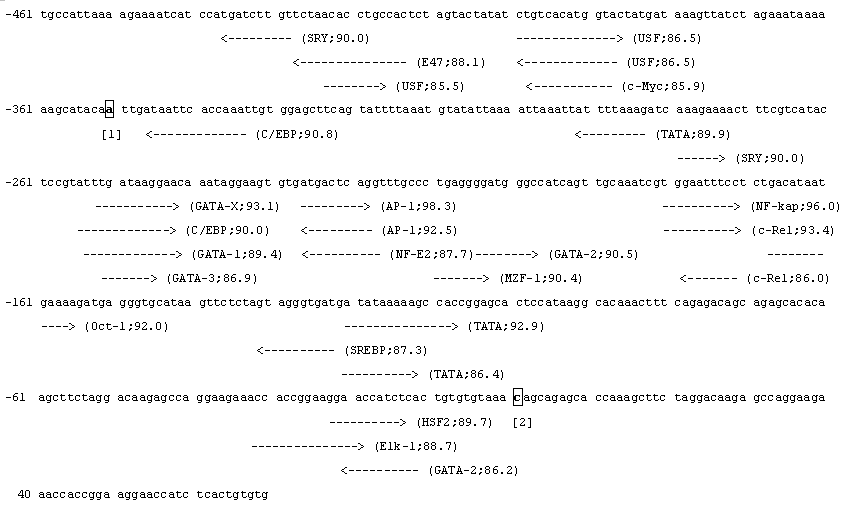


**Supplementary figure 2.** DNA sequences of the IL-8 promoter extending through bp –461 upstream of the transcription factors are underlined (Transcription factor name;score). The rs4073T>A SNP shows no transcription factor rs4073T>A, box [1]). The starting nucleotide of transcription is indicated by the number 1 (+1, box [2]).
